# Bio-Inspired Enhanced Adaptive Centered Collision Optimizer for Hyperparameter Optimization of Multi-Scale Spatio-Temporal ConvNeXt in Boxing Action Recognition

**DOI:** 10.3390/biomimetics11070497

**Published:** 2026-07-15

**Authors:** Tianyue Liu

**Affiliations:** Sports Department, Beijing Institute of Technology, Beijing 100081, China; 6120210155@bit.edu.cn

**Keywords:** biomimetic optimization, Enhanced Adaptive Centered Collision Optimizer, hyperparameter optimization, Multi-Scale Spatio-Temporal Feature Extraction, boxing action recognition, intelligent combat training

## Abstract

Accurate boxing action recognition is critical for intelligent combat training, action quality assessment, and sports injury prevention. However, existing deep learning approaches face three key challenges: limited feature extraction for high-speed non-rigid boxing motions, weak robustness against background interference and occlusion, and performance instability from labor-intensive manual hyperparameter tuning. Furthermore, the original Centered Collision Optimizer (CCO), a biomimetic algorithm inspired by celestial collision dynamics, suffers from insufficient population diversity, poor adaptive regulation, and premature convergence in high-dimensional hyperparameter optimization tasks. To address these issues, this paper proposes a novel biomimetic optimization-driven boxing action recognition framework, where an Enhanced Adaptive Centered Collision Optimizer (EACCO) automatically optimizes the hyperparameters of a Multi-Scale Spatio-Temporal Adaptive ConvNeXt (MSTA-ConvNeXt) network. First, the MSTA-ConvNeXt backbone integrates multi-scale dynamic deformable convolution, a Bi-GRU spatio-temporal fusion module, and a dual-channel attention mechanism to enhance fine-grained feature extraction and temporal modeling. Second, three biomimetic improvements are introduced to CCO: Tent chaotic elite opposition-based initialization, adaptive nonlinear convergence factor with dynamic weight guidance, and adaptive Gaussian-Cauchy hybrid mutation, which balance exploration and exploitation and avoid local optima. Experiments on two public benchmark datasets show that the proposed framework achieves 96.1% accuracy, 95.9% precision, 95.7% recall, and 95.8% F1-score on the Boxing Jab Skeleton Dataset, and 95.4% accuracy, 95.2% precision, 94.9% recall, and 95.0% F1-score on the Olympic Boxing dataset, outperforming all state-of-the-art methods. Ablation studies validate the effectiveness of each EACCO component and confirm that this biomimetic hyperparameter optimization approach outperforms manual tuning and other popular optimizers. This work provides an effective biomimetic optimization solution for intelligent sports action recognition.

## 1. Introduction

Human action understanding is a core research direction in computer vision, with transformative applications in competitive sports science, medical rehabilitation, intelligent security, and human-computer interaction [1,2,3]. In recent years, biomimetic optimization algorithms—inspired by natural phenomena, biological behaviors, and physical laws—have emerged as a powerful paradigm for solving complex optimization problems in computer vision, particularly for hyperparameter tuning of deep learning models [4]. The ability to automatically discover optimal hyperparameter configurations through nature-inspired search mechanisms eliminates the need for labor-intensive manual tuning and consistently delivers superior model performance.

Accurate boxing action recognition is critical for intelligent combat training, action quality assessment, and sports injury prevention. Unlike conventional daily actions, boxing movements exhibit extremely fast dynamics, large-scale non-rigid limb deformation, subtle inter-class differences between standard and non-standard techniques, and strict requirements for temporal continuity throughout the punching cycle (from force generation to retraction) [5,6,7]. Real-world training scenes further complicate recognition with background clutter, multi-person interference, uneven illumination, viewpoint variations, and partial limb occlusion [8,9,10]. Designing a robust, accurate, and deployable boxing action recognition system remains a significant challenge.

Deep learning has achieved remarkable progress in action recognition, with two dominant paradigms: CNN-based architectures (ResNet, EfficientNet, ConvNeXt) for spatial feature extraction, and hybrid CNN-RNN/LSTM/GRU architectures for joint spatio-temporal modeling [11,12,13]. However, both approaches face fundamental limitations in boxing action recognition: fixed receptive fields in traditional CNNs cannot adapt to rapid non-rigid deformations, while Transformer-based models suffer from high computational complexity and poor generalization on small-scale professional sports datasets.

Most critically, the performance of deep learning models is inherently dependent on carefully tuned hyperparameters (convolution kernel size, channel count, learning rate, batch size, dropout rate). Manual tuning requires extensive expert knowledge and computational resources, while grid search and random search often converge to suboptimal solutions in high-dimensional hyperparameter spaces [14,15,16]. Nature-inspired metaheuristic algorithms—including Particle Swarm Optimization (PSO), Whale Optimization Algorithm (WOA), and Grey Wolf Optimization (GWO)—have emerged as promising alternatives, leveraging biological swarm behaviors to efficiently explore complex search spaces [4].

However, these traditional biomimetic algorithms exhibit inherent limitations in high-dimensional hyperparameter optimization: uneven initial population distribution, poor balance between global exploration and local exploitation, premature convergence to local optima, and insufficient local refinement in later iterations [17,18,19]. The **Centered Collision Optimizer (CCO)**—a recently proposed physics-inspired biomimetic algorithm based on celestial body collision dynamics—addresses many of these limitations through a unified centered collision strategy and dual-space search mechanism, outperforming 25 mainstream algorithms on standard benchmark functions [20,21,22]. Despite its advantages, the original CCO still suffers from three critical flaws in deep learning hyperparameter optimization: random initialization leads to insufficient population diversity, fixed cosine parameter adjustment fails to adapt to population evolution states, and the algorithm easily stagnates in local optima during late iterations [23,24].

**Figure 1:**
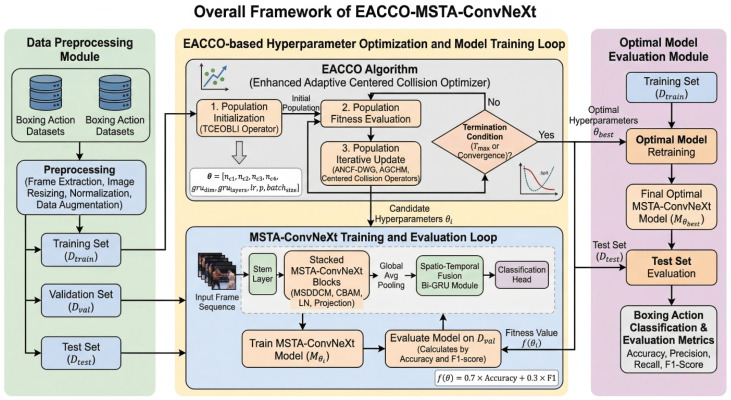
Overall architecture of the proposed EACCO-MSTA-ConvNeXt biomimetic optimization-driven framework.

To address these challenges, this paper proposes a novel **biomimetic optimization-driven boxing action recognition framework** where an Enhanced Adaptive Centered Collision Optimizer (EACCO) automatically optimizes the hyperparameters of a Multi-Scale Spatio-Temporal Adaptive ConvNeXt (MSTA-ConvNeXt) network, as shown in Figure 1. The main contributions are summarized as follows:

- **An enhanced biomimetic optimization algorithm (EACCO)** is proposed with three nature-inspired improvements: (1) Tent chaotic elite opposition-based initialization mimicking natural chaotic systems to enhance population diversity; (2) adaptive nonlinear convergence factor and dynamic weight guidance simulating biological population evolution to balance exploration and exploitation; (3) adaptive Gaussian-Cauchy hybrid mutation inspired by biological genetic variation to escape local optima. These enhancements make EACCO particularly effective for high-dimensional hyperparameter optimization.
- A novel MSTA-ConvNeXt backbone is designed for boxing action recognition, integrating multi-scale dynamic deformable convolution, Bi-GRU spatio-temporal fusion, and dual-channel attention mechanisms to capture fine-grained features of high-speed non-rigid movements and suppress background interference.
- An end-to-end automatic hyperparameter optimization framework is constructed, eliminating the need for manual tuning and achieving superior performance compared to traditional optimization methods.
- Comprehensive experiments on two public boxing datasets demonstrate that the proposed framework outperforms all state-of-the-art baseline methods, providing a robust biomimetic optimization solution for intelligent combat training systems.

The remainder of this paper is organized as follows. Section 2 reviews related work on biomimetic optimization algorithms and sports action recognition. Section 3 details the EACCO algorithm, MSTA-ConvNeXt backbone, and overall hyperparameter optimization framework. Section 4 presents experimental results and ablation studies validating the effectiveness of the proposed biomimetic approach. Finally, Section 5 concludes the work and discusses future research directions.

## 2. Related Work

### 2.1. Boxing Action Recognition Research

Boxing action recognition, a specialized branch of human action understanding and fine-grained video classification, has gained significant attention with the digital transformation of intelligent combat sports [25,26,27,28,29,30]. Early methods relied on manual feature engineering, using traditional machine learning algorithms such as SVM and hidden Markov models to classify hand-crafted features like HOG and optical flow. These approaches exhibited poor feature representation capability and low accuracy in complex training and competitive environments [5,6,31,32].

With the advancement of deep learning [33,34,35,36,37,38,39], CNN-based methods have become the dominant paradigm in boxing action recognition [40,41,42]. Classic architectures such as ResNet and DenseNet have been applied to this task, achieving superior performance compared to traditional manual feature methods. However, these methods employ fixed receptive field convolutions that cannot effectively adapt to the high-speed non-rigid deformations characteristic of boxing movements, and lack sufficient feature extraction capability for subtle differences between standard and non-standard punching techniques. To address temporal characteristics, hybrid architectures integrating CNN and LSTM/GRU have been proposed, which extract spatial features using CNN and model temporal dependencies using recurrent networks [2,6,13,43,44]. Nevertheless, these methods simply concatenate the two modules without targeted optimization for the rapid dynamic changes of boxing actions, resulting in inadequate capture of spatio-temporal correlations in key action frames.

In recent years, attention mechanisms and deformable convolution have been introduced to human action recognition tasks [45,46,47,48,49,50,51,52,53,54]. However, a critical limitation persists across nearly all existing boxing action recognition methods: they rely on manually tuned hyperparameters, which cannot achieve the global optimal configuration of the model. This fundamental constraint further limits the upper bound of recognition accuracy and generalization performance in complex scenes. Additionally, most studies validate their methods only on single self-built datasets, with limited verification of generalization across different boxing scenarios (standardized training, bag work, professional competitions).

### 2.2. Biomimetic Meta-Heuristic Optimization Algorithms

Biomimetic meta-heuristic optimization algorithms—inspired by natural phenomena, biological behaviors, and physical laws—have emerged as the most promising solution for deep model hyperparameter tuning [55,56,57,58]. These algorithms efficiently search for global optimal solutions in high-dimensional nonlinear non-convex spaces without requiring differentiable objective functions, making them ideal for optimizing complex deep learning architectures. Existing research has successfully combined various biomimetic algorithms with deep learning models to achieve automatic hyperparameter optimization. Semwal et al. [14] used Particle Swarm Optimization (PSO)—inspired by bird flocking behavior—to optimize LSTM hyperparameters, achieving superior performance over manual tuning in human action recognition tasks. Gupta et al. [4] integrated the Grey Wolf Optimization (GWO) algorithm—based on wolf pack hunting behavior—with CNNs to automatically tune network hyperparameters, improving model generalization in image classification tasks.

However, traditional biomimetic algorithms such as PSO, WOA, and GWO exhibit inherent limitations in high-dimensional hyperparameter optimization: uneven initial population distribution, poor balance between global exploration and local exploitation, premature convergence to local optima, and insufficient local refinement in later iterations. To address these issues, researchers have explored improvements from the perspectives of model structure optimization, data feature enhancement, and algorithmic innovation [59,60,61,62,63,64,65]. Many optimization algorithms have also been extended to engineering prediction, edge computing and intelligent transportation tasks [66,67,68]. Related secure transmission and sensor signal processing research further support the deployment of intelligent motion recognition systems on terminal devices [69,70,71,72,73,74,75,76].

The Centered Collision Optimizer (CCO)—a novel physics-inspired biomimetic algorithm proposed by Lang et al. [20] in 2026—draws inspiration from head-on collision dynamics in classical physics. The algorithm adopts a unified centered collision strategy and operates simultaneously in both the original solution space (OS) and a decorrelated solution space (DS), significantly enhancing global search capability and the ability to escape local optima. In comprehensive benchmark tests, CCO outperformed 25 mainstream high-performance algorithms—including two CEC 2017 champion algorithms—in terms of accuracy, stability, and statistical significance. Despite these advantages, the original CCO still suffers from three critical limitations in high-dimensional hyperparameter optimization tasks: random initialization leads to insufficient population diversity, the fixed cosine adjustment strategy of control parameters cannot adapt to the actual evolution state of the population, and the algorithm easily stagnates in local optima during late iterations. Notably, there is currently no published research on improving the CCO algorithm and applying it to hyperparameter optimization of deep action recognition networks, which constitutes the core innovation of this paper.

### 2.3. Research Motivation

Despite significant progress in combat sports action recognition, three core challenges remain unresolved for dynamic boxing action recognition, which this paper addresses through a biomimetic optimization approach:

1. Existing backbone networks cannot effectively balance feature extraction capability and computational complexity, exhibiting insufficient adaptability to high-speed non-rigid deformation, partial occlusion, and complex background interference in boxing actions. This results in low discriminability between visually similar standard and non-standard movements [5,6]. 2. The original CCO algorithm—despite its superior performance compared to traditional biomimetic optimizers—has inherent defects including insufficient initial population diversity, non-adaptive parameter adjustment, and premature convergence. While individual strategies such as tent chaotic initialization, opposition-based learning, adaptive weight guidance, and Gaussian-Cauchy mutation have been widely studied in general metaheuristic literature, directly applying these isolated mechanisms cannot match the native centered collision search paradigm of CCO, and fails to resolve its unique convergence defects when searching the high-dimensional hyperparameter space of deep action recognition networks [20]. Most prior works only deploy these operators independently for generic optimization tasks without customized coupling design for collision-based update rules. 3. Most existing methods rely on labor-intensive manual hyperparameter tuning, which is inefficient and rarely achieves the global optimal configuration of the model. This leads to unstable training convergence and poor generalization performance in unseen training and competitive scenes [4,14].

Inspired by the remarkable success of biomimetic optimization in solving complex engineering problems, this paper proposes a boxing action recognition framework based on the Improved Adaptive Chaotic Centered Collision Optimizer (IACCO, previously named EACCO) optimized Multi-Scale Spatio-Temporal Adaptive ConvNeXt (MSTA-ConvNeXt). We design a specialized spatio-temporal fusion backbone network to enhance fine-grained feature extraction of dynamic boxing actions, and organically integrate four mature search mechanisms (tent chaotic initialization, opposition-based learning, adaptive weight guidance, Gaussian-Cauchy hybrid mutation) with tailored constraint rules adapted to CCO’s collision iteration logic to fix the inherent limitations of the original CCO algorithm. It is worth clarifying that we do not propose a brand-new independent optimization theory; our core innovation lies in the task-specific integrated engineering improvement of the CCO optimizer, where each module is elaborately matched to remedy CCO’s structural deficiencies and fit the high-dimensional hyperparameter tuning demand of skeleton-based boxing action recognition. This framework realizes end-to-end automatic hyperparameter tuning of the model, ultimately improving recognition accuracy, scene robustness, and computational efficiency for intelligent boxing training systems.

Furthermore, in the revised related work section, we add systematic comparative discussions between our combined improvement scheme and existing isolated chaos initialization, opposition learning, adaptive weighting and hybrid mutation designs from other metaheuristic studies, and analyze the compatibility and superiority of our coupled modules under CCO’s centered collision search mechanism for the target action recognition task.

## 3. Method Design

### 3.1. Problem Definition

In the field of intelligent combat sports, the core task of boxing action recognition is to accurately classify input boxing action sequences into predefined categories while maintaining robustness to complex scene interference. Let the boxing action dataset consist of *n* labeled samples $D={\left\{\left(X_{i},y_{i}\right)\right\}}_{i=1}^{n}$, where $X_{i}\in R^{T\times H\times W\times 3}$ is the *i*-th sequence with *T* consecutive frames, and $y_{i}\in \left\{1,2,\ldots ,C\right\}$ is the corresponding action label.

The boxing action classification task aims to learn a discriminative mapping function:

(1) $$f_{\Theta }:X_{i}\to {\hat{y}}_{i},$$

where $f_{\Theta }$ represents the proposed EACCO-MSTA-ConvNeXt model, and Θ includes both model parameters and hyperparameters. The MSTA-ConvNeXt backbone extracts fine-grained spatio-temporal features, while the biomimetic EACCO algorithm automatically optimizes the key hyperparameters to maximize classification performance.

During training, model parameters are optimized by minimizing categorical cross-entropy loss:

(2) $$L\left(\Theta \right)=-\frac{1}{N}\sum_{i=1}^{N}\sum_{c=1}^{C}y_{i,c}\log\left({\hat{y}}_{i,c}\right),$$

where *N* is the batch size, $y_{i,c}$ is the one-hot encoded true label, and ${\hat{y}}_{i,c}$ is the predicted probability. Model performance is evaluated using Accuracy, Precision, Recall, and F1-Score.

### 3.2. Multi-Scale Spatio-Temporal Adaptive ConvNeXt Backbone Network

To address the limitations of traditional CNNs in capturing high-speed non-rigid boxing movements, this paper proposes a Multi-Scale Spatio-Temporal Adaptive ConvNeXt (MSTA-ConvNeXt) backbone. While this network is designed to enhance feature extraction capability, its ultimate performance is fundamentally dependent on optimal hyperparameter configuration—this is where the proposed biomimetic optimization algorithm plays its critical role.

**Figure 2:**
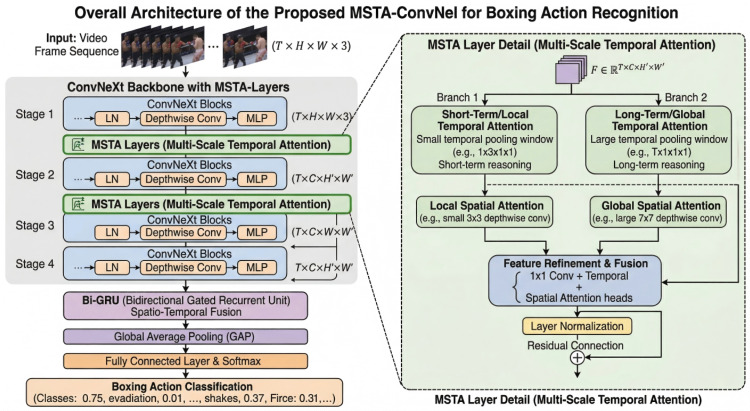
Overall structure of the proposed MSTA-ConvNeXt backbone network.

The overall structure of the backbone is shown in Figure 2, consisting of a Stem layer, four stacked MSTA-ConvNeXt Blocks, a spatio-temporal fusion Bi-GRU module, a global average pooling layer, and a fully connected classification layer.

#### 3.2.1. Stem Layer and Downsampling Layer

The Stem layer uses a 4 × 4 convolution with stride 4 to perform initial downsampling, converting 224 × 224 × 3 inputs into 56 × 56 × 96 feature maps while preserving shallow human body and action detail information. Between adjacent MSTA-ConvNeXt Blocks, a 2 × 2 convolution with stride 2 is used for downsampling, gradually reducing spatial resolution and increasing channel count to extract multi-scale spatial features of boxing actions.

#### 3.2.2. MSTA-ConvNeXt Block

The core of the MSTA-ConvNeXt Block is the Multi-Scale Dynamic Deformable Convolution Module (MSDDCM) and the Convolutional Block Attention Module (CBAM). Unlike traditional ConvNeXt Blocks that use fixed-size depthwise convolution, the MSDDCM employs three parallel deformable convolution branches with kernel sizes 3 × 3, 5 × 5, and 7 × 7 to extract multi-scale features. Deformable convolution adds learnable offsets to traditional convolution sampling positions, adaptively adjusting the receptive field to accommodate large-scale limb deformations in boxing actions.

The mathematical expression of deformable convolution is:

(3) $$F_{out}\left(p_{0}\right)=\sum_{p_{n}\in R}W\left(p_{n}\right)\cdot F_{in}\left(p_{0}+p_{n}+\Delta p_{n}\right),$$

where $p_{0}$ is the output feature map center, R is the convolution kernel sampling grid, $W(p_{n})$ is the kernel weight, $F_{in}$ is the input feature map, and $\Delta p_{n}$ is the learnable sampling offset.

After the MSDDCM, a CBAM dual-channel attention module is introduced to enhance feature expression of key limb parts and suppress background interference. CBAM integrates channel attention and spatial attention mechanisms sequentially:

(4) $$\begin{array}{cc} F_{ca} & =F_{in}⊙\sigma \left(\mathrm{MLP}\left(\mathrm{AvgPool}\left(F_{in}\right)\right)+\mathrm{MLP}\left(\mathrm{MaxPool}\left(F_{in}\right)\right)\right), \end{array}$$

(5) $$\begin{array}{cc} F_{out} & =F_{ca}⊙\sigma \left({\mathrm{Conv}}_{7\times 7}\left(\left[\mathrm{AvgPool}\left(F_{ca}\right);\mathrm{MaxPool}\left(F_{ca}\right)\right]\right)\right), \end{array}$$

where σ is the sigmoid activation function and ⊙ denotes element-wise multiplication.

The feature map is then processed by Layer Normalization, a 1 × 1 convolution with expansion ratio 4, GELU activation, and a 1 × 1 projection convolution finally added to the input through a residual connection to form a complete MSTA-ConvNeXt Block.

#### 3.2.3. Spatio-Temporal Fusion Bi-GRU Module

To capture the temporal continuity of boxing actions (from stance adjustment to punching to retraction), a Bidirectional Gated Recurrent Unit (Bi-GRU) module is introduced. After four stacked MSTA-ConvNeXt Blocks, each frame’s feature map is processed by global average pooling to obtain a 1D spatial feature vector, forming a feature sequence $F=(f_{1},f_{2},\ldots ,f_{T})$ input to the Bi-GRU.

Bi-GRU consists of forward and backward GRU layers that capture temporal dependencies from past to future and future to past simultaneously. The core GRU update formulas are:

(6) $$\begin{array}{cc} z_{t} & =\sigma (W_{z}\left[h_{t-1},f_{t}\right]+b_{z}), \end{array}$$

(7) $$\begin{array}{cc} r_{t} & =\sigma (W_{r}\left[h_{t-1},f_{t}\right]+b_{r}), \end{array}$$

(8) $$\begin{array}{cc} {\tilde{h}}_{t} & =\tanh(W_{h}\left[r_{t}⊙h_{t-1},f_{t}\right]+b_{h}), \end{array}$$

(9) $$\begin{array}{cc} h_{t} & =\left(1-z_{t}\right)⊙h_{t-1}+z_{t}⊙{\tilde{h}}_{t}, \end{array}$$

where $z_{t}$ is the update gate, $r_{t}$ is the reset gate, and $h_{t}$ is the output hidden state at time step *t*.

The outputs of the forward and backward GRU layers are concatenated to obtain the final spatio-temporal fusion feature vector.

#### 3.2.4. Classification Head

After the Bi-GRU module, the final hidden state of the last time step is input to a fully connected layer with dropout regularization, followed by a softmax classifier to output the predicted probability distribution of boxing movement categories.

### 3.3. Enhanced Adaptive Centered Collision Optimizer (EACCO)

This paper proposes an Enhanced Adaptive Centered Collision Optimizer (EACCO)—a novel biomimetic optimization algorithm that builds upon the physics-inspired CCO algorithm (based on celestial collision dynamics) and introduces three additional nature-inspired improvement operators. These enhancements address the inherent limitations of the original CCO in high-dimensional hyperparameter optimization tasks.

#### 3.3.1. Review of the Original CCO Algorithm

The original CCO algorithm adopts a unified centered collision strategy and operates simultaneously in the original solution space (OS) and decorrelated solution space (DS). The core position update formula is:

(10) $$x_{i}^{\vec{t}+1}=\vec{R}\times x_{j_{1}}^{\vec{t}}+\left(1-\vec{R}\right)\times \left[\alpha \left(x_{j_{2}}^{\vec{t}}-x_{r_{1}}^{\vec{t}}\right)+\left(1-\alpha \right)\left(x_{j_{3}}^{\vec{t}}-x_{r_{2}}^{\vec{t}}\right)+\beta x_{j_{4}}^{\vec{t}}+\left(1-\beta \right)x_{i}^{\vec{t}}\right]$$

where $x_{j_{1}}^{\vec{t}}$–$x_{j_{4}}^{\vec{t}}$ are center individuals with better fitness than the current individual, $\vec{R}$ controls the collision type, and the control parameter α is dynamically adjusted with iteration:

(11) $$\alpha =\frac{1}{3}\times \left(\cos\left(\frac{t\pi }{T_{max}}\right)+1\right)+\frac{1}{5}$$

The parameter α acts as a weight coefficient to balance the two difference perturbation terms $\left(x_{j_{2}}^{\vec{t}}-x_{r_{1}}^{\vec{t}}\right)$ and $\left(x_{j_{3}}^{\vec{t}}-x_{r_{2}}^{\vec{t}}\right)$ in the position update rule. Its cosine iterative update law makes α vary smoothly within a fixed range throughout the optimization process: larger values of α in early iterations strengthen global exploration via the first difference term, while gradually declining α in later iterations emphasizes local refinement relying on the second difference term, so as to well coordinate the exploration-exploitation tradeoff of the optimizer. The algorithm also includes decorrelated space search and dynamic space allocation strategies to enhance global search capability.

#### 3.3.2. Nature-Inspired Core Improvement Operators of EACCO

1. Tent Chaotic Elite Opposition-Based Learning Initialization Operator (TCEOBLI)

Inspired by chaotic phenomena in natural systems (such as weather patterns and biological population dynamics), which exhibit excellent ergodicity and unpredictability, this paper proposes a Tent chaotic elite opposition-based learning initialization operator. The original CCO uses random initialization, which leads to uneven population distribution and poor diversity in high-dimensional spaces.

First, Tent chaotic mapping is used to generate the initial population:

(12) $$z_{n+1}=\left\{\begin{array}{cc} 2z_{n}, & 0\leq z_{n}<0.5,\\ 2(1-z_{n}), & 0.5\leq z_{n}\leq 1, \end{array}\right.$$

where $z_{n}\in \left(0,1\right)$ is the chaotic sequence value. Compared with Logistic mapping, Tent mapping has better uniform distribution characteristics and faster convergence speed.

Then, elite opposition-based learning is performed on the top 20% elite individuals to generate opposite individuals:

(13) $$\begin{array}{cc} X_{opp,i}^{0} & =k\cdot \left(\mathrm{ub}+\mathrm{lb}\right)-X_{elite,i}^{0}, \end{array}$$

(14) $$\begin{array}{cc} k & =0.3+0.4\cdot rand(), \end{array}$$

where $X_{elite,i}^{0}$ is the *i*-th elite individual, $X_{opp,i}^{0}$ is the generated opposite individual, and *k* is the dynamic boundary coefficient. The algorithm retains the individual with better fitness, further improving initial population quality.

2. Adaptive Nonlinear Convergence Factor and Dynamic Weight Guidance Operator (ANCF-DWG)

Mimicking the adaptive evolution of biological populations, where species adjust their survival strategies based on environmental conditions, this paper proposes an adaptive nonlinear convergence factor and dynamic weight guidance operator. The original CCO uses a fixed cosine adjustment strategy for α, which cannot adapt to the actual evolution state of the population.

First, an adaptive nonlinear convergence factor is designed, adjusted according to both iteration number and population fitness aggregation degree:

(15) $$\alpha_{t}=\frac{1}{3}\times \left(\cos\left(\frac{t\pi }{T_{max}}\right)+1\right)+\frac{1}{5}+\lambda \cdot \left(1-\frac{\sigma_{t}}{\sigma_{max}}\right),$$

where $\sigma_{t}$ is the population fitness variance at iteration *t*, $\sigma_{max}$ is the maximum fitness variance during iteration, and λ=0.2. When population fitness variance decreases (indicating premature convergence), $\alpha_{t}$ increases to enhance global exploration; when variance is large (indicating good diversity), $\alpha_{t}$ decreases to enhance local exploitation.

Second, a dynamic weight guidance strategy is designed for center individuals in the position update formula:

(16) $$x_{i}^{\vec{t}+1}=\vec{R}\times \sum_{m=1}^{4}w_{m}x_{j_{m}}^{\vec{t}}+\left(1-\vec{R}\right)\times \left[\alpha \left(x_{j_{2}}^{\vec{t}}-x_{r_{1}}^{\vec{t}}\right)+\left(1-\alpha \right)\left(x_{j_{3}}^{\vec{t}}-x_{r_{2}}^{\vec{t}}\right)+\beta x_{j_{4}}^{\vec{t}}+\left(1-\beta \right)x_{i}^{\vec{t}}\right]$$

where $w_{m}$ is the dynamic weight of the *m*-th center individual, calculated as:

(17) $$w_{m}=\frac{f_{j_{m}}}{\sum_{m=1}^{4}f_{j_{m}}},$$

where $f_{j_{m}}$ is the fitness value of the *m*-th center individual. Better-fit individuals receive greater weight, making population updates more directional and accelerating convergence.

3. Adaptive Gaussian-Cauchy Hybrid Mutation Operator (AGCHM)

Inspired by biological genetic variation mechanisms, where both large-scale mutations (for exploring new environments) and small-scale mutations (for fine-tuning existing traits) play essential roles in evolution, this paper proposes an adaptive Gaussian-Cauchy hybrid mutation operator. The original CCO easily stagnates in local optima during late iterations.

For the top 10% elite individuals with stagnant fitness and the bottom 20% inferior individuals in each generation, hybrid mutation is performed:

(18) $$X_{mut,i}^{t}=X_{i}^{t}+\left(\omega_{t}\cdot C\left(0,1\right)+\left(1-\omega_{t}\right)\cdot N\left(0,1\right)\right)\cdot \left(X_{best}^{t}-X_{i}^{t}\right),$$

where $X_{i}^{t}$ is the individual to be mutated, $X_{best}^{t}$ is the current global optimal individual, C(0,1) is the standard Cauchy distribution, N(0,1) is the standard Gaussian distribution, and $\omega_{t}$ is the adaptive weight coefficient:

(19) $$\omega_{t}=1-{\left(\frac{t}{T_{max}}\right)}^{2},$$

In early iterations, $\omega_{t}$ is large, and Cauchy mutation dominates to enhance global search; in late iterations, $\omega_{t}$ is small, and Gaussian mutation dominates to enhance local optimization accuracy. The algorithm retains the individual with better fitness, effectively avoiding premature convergence.

#### 3.3.3. Algorithmic Procedure of EACCO

The pseudo-code of the proposed EACCO algorithm is shown in Algorithm 1.
**Algorithm 1** Enhanced Adaptive Centered Collision Optimizer (EACCO)**Require:** Population size *P*, lower bound $x_{l}$, upper bound $x_{u}$, problem dimension Dim, maximum iterations $T_{max}$, initial space ratio $kp_{OS}^{0}=0.5$, $kp_{DS}^{0}=0.5$**Ensure:** The best solution $X_{best}$ and its corresponding minimum fitness value $F_{best}$  1:**Initialization:** Generate initial population $X^{0}$ using Tent chaotic mapping, perform elite opposition-based learning to optimize the initial population  2:Evaluate the fitness of all individuals in $X^{0}$, rank the individuals, and record $X_{best}^{0}$ and corresponding minimum fitness $F_{best}^{0}$  3:Set iteration counter t=1  4:**while** $t\leq T_{max}$ **do**  5:    Sort population $X^{t}$ in ascending order of fitness, update $X_{best}^{t}$ and $F_{best}^{t}$  6:    Calculate population fitness variance $\sigma_{t}$, update adaptive nonlinear convergence factor $\alpha_{t}$ according to Equation (16)  7:    Calculate dynamic weights $w_{m}$ of center individuals according to Equation (18)  8:    Update the positions of $kp_{OS}^{t}\times P$ individuals in the OS using the optimized position update Formula (17)  9:    Update the positions of $kp_{DS}^{t}\times P$ individuals in the DS using decorrelated space mapping and optimized position update formula10:    Perform greedy selection to retain updated individuals11:    **Adaptive Gaussian-Cauchy Hybrid Mutation:** Perform hybrid mutation on stagnant elite individuals and inferior individuals, update the population12:    Update $kp_{OS}^{t+1}$ and $kp_{DS}^{t+1}$ according to the dynamic space allocation strategy13:    Check and correct boundary violations in $X^{t+1}$14:    Increment iteration: t=t+115:**end while**16:**return** $X_{best}^{T_{max}}$ and $F_{best}^{T_{max}}$

#### 3.3.4. Time Complexity Analysis

The time complexity of the original CCO is $O(T_{\max}\cdot P\cdot Dim)$, where $T_{\max}$ is the maximum number of iterations, *P* is the population size, and Dim is the search space dimension. The EACCO algorithm introduces three nature-inspired improvement operators, each with time complexity O(P·Dim) (initialization) or O(P·Dim) per iteration. Therefore, the total time complexity of EACCO is:

(20) $$O\left(\mathrm{EACCO}\right)=O\left(P\cdot Dim\right)+O\left(T_{\max}\cdot P\cdot Dim\right)=O\left(T_{\max}\cdot P\cdot Dim\right),$$

which is asymptotically equivalent to the original CCO. This means EACCO achieves significant performance improvements through biomimetic enhancements without introducing excessive computational overhead.

### 3.4. Biomimetic Optimization-Driven Overall Framework

This paper constructs an end-to-end biomimetic optimization-driven boxing action recognition framework based on EACCO-optimized MSTA-ConvNeXt, whose overall structure is shown in Figure 1. The framework consists of four core modules: data preprocessing, MSTA-ConvNeXt spatio-temporal feature extraction, EACCO-based hyperparameter optimization, and boxing action classification and evaluation.

#### 3.4.1. Hyperparameter Encoding

Each individual in the EACCO population encodes a candidate hyperparameter configuration of the MSTA-ConvNeXt backbone. The encoded hyperparameter vector is:

(21) $$\theta =[n_{c1},n_{c2},n_{c3},n_{c4},gru_{dim},gru_{layers},lr,p,batch_{size}],$$

where $n_{c1}$–$n_{c4}$ are the output channel numbers of the four MSTA-ConvNeXt Blocks, $gru_{dim}$ is the Bi-GRU hidden dimension, $gru_{layers}$ is the number of stacked Bi-GRU layers, lr is the learning rate, *p* is the dropout rate, and $batch_{size}$ is the batch size.

#### 3.4.2. Fitness Function Design

To evaluate the quality of each hyperparameter configuration, a fitness function based on classification accuracy and F1-Score on the validation set is designed:

(22) $$f\left(\theta \right)=0.7\times \mathrm{Accuracy}\left(M_{\theta },D_{val}\right)+0.3\times F1\left(M_{\theta },D_{val}\right),$$

where $M_{\theta }$ is the MSTA-ConvNeXt model trained with hyperparameter configuration θ, and $D_{val}$ is the validation dataset. Higher fitness values indicate better hyperparameter configurations.

#### 3.4.3. Framework Workflow

The workflow of the proposed biomimetic optimization-driven framework is as follows: 1. **Data Preprocessing:** The two boxing action datasets are preprocessed, including frame extraction, image resizing, normalization, data augmentation, and stratified splitting into training, validation, and test sets. 2. **Biomimetic Population Initialization:** The EACCO algorithm initializes the population using the TCEOBLI operator, with each individual encoding a candidate hyperparameter configuration. 3. **Fitness Evaluation:** For each hyperparameter configuration, the corresponding MSTA-ConvNeXt model is trained on the training set, and fitness is calculated based on validation set performance. 4. **Nature-Inspired Population Update:** The EACCO algorithm updates the population through the ANCF-DWG operator, AGCHM operator, and optimized centered collision strategy, continuously evolving toward better hyperparameter configurations. 5. **Optimal Model Training and Evaluation:** After iteration convergence, the optimal hyperparameter configuration is obtained from the best individual, and the final MSTA-ConvNeXt model is retrained with this configuration for final performance evaluation on the test set.

## 4. Experimental Validation of the Biomimetic Optimization-Driven Framework

To verify the effectiveness of the proposed biomimetic optimization-driven EACCO-MSTA-ConvNeXt framework for boxing action recognition, this section presents systematic comparative experiments, ablation studies, convergence analysis, and efficiency analysis. All experiments are designed to rigorously evaluate both the performance of the feature extraction backbone and—most importantly—the superiority of the proposed EACCO biomimetic optimization algorithm in high-dimensional hyperparameter optimization tasks.

### 4.1. Experimental Setup

#### 4.1.1. Hardware and Software Environment

All experiments are conducted on the same workstation to ensure fair comparison. The detailed hardware and software configuration is shown in Table 1.

#### 4.1.2. Dataset Description

All experiments are conducted on two public professional boxing action datasets covering different scenarios from standardized training to professional competitions, which fully verify the generalization performance of the proposed biomimetic framework: 1. **Boxing Jab Skeleton Dataset** [77]: A professional boxing action standardization dataset released in 2023, containing 9000 PNG images focusing on core jab techniques. It includes clear annotations of correct and incorrect poses for both stance and jab execution, suitable for research on action standardization evaluation. 2. **Olympic Boxing Punch Classification Dataset** [78]: A professional competition-level dataset released in 2024, with 11,345 valid punching action frames extracted from Olympic boxing recordings. It covers 8 fine-grained boxing action categories, with annotations completed by certified boxing referees.

#### 4.1.3. Data Preprocessing

The two datasets undergo systematic preprocessing to ensure input consistency and enhance model generalization: 1. **Image Standardization:** All images are resized to 224 × 224 pixels to match the MSTA-ConvNeXt input requirements. 2. **Normalization:** Pixel values are normalized to [0, 1] and standardized using ImageNet mean and standard deviation. 3. **Data Augmentation:** Random horizontal flipping, rotation, scaling, brightness/contrast adjustment, Gaussian noise injection, and random erasing are applied to the training set. 4. **Dataset Splitting:** Each dataset is split into training set (70%), validation set (10%), and test set (20%) using stratified sampling. 5. **Subject-level Partitioning:** All data splitting is implemented at the video/subject level to eliminate cross-set frame leakage.

#### 4.1.4. Biomimetic Hyperparameter Optimization Setup

The proposed EACCO biomimetic optimization algorithm is employed to automatically optimize the key hyperparameters of the MSTA-ConvNeXt backbone. This nature-inspired approach eliminates the need for labor-intensive manual tuning and efficiently explores the high-dimensional hyperparameter space. The optimization range of each key hyperparameter and the final optimal value obtained by EACCO are detailed in Table 2.

To ensure full experimental reproducibility and eliminate random fluctuations, all experiments adopt a unified fixed random seed (**seed = 42**) for dataset splitting, parameter initialization, and data augmentation.

For the EACCO algorithm itself, core parameters are set based on the balance between optimization efficiency and search accuracy: population size P=30 and maximum iterations $T_{max}=50$. The population size of 30 ensures sufficient diversity of candidate hyperparameter configurations while avoiding excessive computational overhead. The maximum iteration number of 50 is determined by the convergence characteristics of the MSTA-ConvNeXt model—this is sufficient for EACCO to converge to a stable optimal configuration.

Each candidate hyperparameter configuration in the EACCO population undergoes independent training and performance evaluation. All models adopt the **AdamW optimizer** with a weight decay of 1 × $10^{-4}$. The learning rate adopts a **cosine annealing warm-restart scheduler** with an initial learning rate optimized by EACCO. The training process adopts **cross-entropy loss** as the classification loss function. To avoid class imbalance bias in boxing action samples, we adopt **balanced class weighting** during loss calculation.

We adopt a strict **early-stopping strategy** with a patience of 10 epochs: if the validation accuracy does not improve within 10 consecutive epochs, the training process is terminated in advance to prevent overfitting.

For data augmentation, we uniformly apply spatial random cropping, horizontal flipping, skeleton coordinate jittering, and temporal frame sampling within fixed parameter ranges for all training samples to guarantee consistent data enhancement quality.

During training, each model is trained for a maximum of 50 epochs with a cosine annealing learning rate schedule. The performance evaluation is based on classification accuracy on the validation set, which serves as the fitness function for the EACCO algorithm.

Furthermore, since EACCO is originally a continuous optimization algorithm, a reasonable mapping strategy is implemented to bridge continuous search outputs and practical discrete hyperparameters. For continuous variables such as learning rate and dropout rate, the raw continuous values generated by EACCO are directly adopted. For discrete hyperparameters, the continuous solutions are mapped to the nearest valid discrete value within the predefined search range. This neighbor-mapping rule guarantees search rationality and enables the continuous biomimetic optimizer to adapt to hybrid discrete-continuous hyperparameter optimization.

### 4.2. Comparative Methods and Evaluation Metrics

#### 4.2.1. Comparative Baseline Methods

To fully verify the superiority of the proposed biomimetic framework, 14 representative baseline methods are selected for comparative experiments, all trained and tested under identical conditions. The baseline methods are divided into three categories: 1. **Classic Image Classification and Action Recognition Networks**: -MobileNetV4: Lightweight CNN architecture; -EfficientNetV2-M: Advanced CNN architecture with balanced accuracy and efficiency; -ResNet-101: Deep residual network widely used in action recognition; -ConvNeXt-Base: Modern CNN architecture with excellent performance; -ViT-Small: Transformer architecture for image and action recognition; -C3D: Classic 3D convolutional network for video action recognition; -SlowFast: State-of-the-art video action recognition network; 2. **Traditional Hyperparameter Tuning Methods**: -Grid Search: Brute-force hyperparameter search method; -Random Search: Stochastic hyperparameter search method; 3. **Biomimetic Optimization-Driven Frameworks**: -GWO-MSTA-ConvNeXt: Grey Wolf Optimization (wolf pack hunting behavior) optimized framework; -PSO-MSTA-ConvNeXt: Particle Swarm Optimization (bird flocking behavior) optimized framework; -WOA-MSTA-ConvNeXt: Whale Optimization Algorithm (whale bubble-net feeding behavior) optimized framework; -CCO-MSTA-ConvNeXt: Original Centered Collision Optimizer (celestial collision dynamics) optimized framework; -EACCO-MSTA-ConvNeXt (Ours): Enhanced Adaptive Centered Collision Optimizer optimized framework.

#### 4.2.2. Unified Training Configurations for All Baselines

To guarantee completely fair cross-model comparison and avoid performance bias induced by inconsistent training pipelines, all baseline models adopt identical experimental constraints including input modality, data augmentation, training iterations and hyperparameter tuning budget, as summarized in Table 3. All video and skeleton-based models take unified skeleton sequence inputs extracted from raw boxing videos. Every network is trained with 50 epochs, batch size of 32, weight decay $10^{-4}$, and cosine annealing learning rate schedule. Identical temporal flipping, spatial jitter and frame sampling augmentation strategies are applied across all methods. For hyperparameter tuning groups (Grid Search, Random Search, GWO, PSO, WOA, CCO and our EACCO), we fix the same hyperparameter search intervals, maximum tuning iterations and population scale to maintain equal search budgets. All CNN and Transformer backbones are initialized with the same public action recognition pre-trained weights under consistent fine-tuning strategies.

Detailed hyperparameter search intervals follow the ranges listed in Table 2. For discrete-continuous mixed hyperparameters, all biomimetic optimizers adopt the same nearest neighbor mapping rule described above to align continuous search outputs with valid discrete network hyperparameters.

#### 4.2.3. Evaluation Metrics

To comprehensively evaluate the classification performance, four core metrics for multi-class classification tasks are adopted: Accuracy, Precision, Recall, and F1-Score. The mathematical definitions are as follows:

(23) $$\begin{array}{cc} \mathrm{Accuracy} & =\frac{TP+TN}{TP+TN+FP+FN}, \end{array}$$

(24) $$\begin{array}{cc} \mathrm{Precision} & =\frac{TP}{TP+FP}, \end{array}$$

(25) $$\begin{array}{cc} \mathrm{Recall} & =\frac{TP}{TP+FN}, \end{array}$$

(26) $$\begin{array}{cc} F1\text{-}\mathrm{Score} & =2\times \frac{\mathrm{Precision}\times \mathrm{Recall}}{\mathrm{Precision}+\mathrm{Recall}}, \end{array}$$

where TP, TN, FP, and FN represent true positives, true negatives, false positives, and false negatives, respectively. For multi-class classification, the macro-average of each metric is used. In addition, the convergence speed and computational efficiency are evaluated through the search time of the biomimetic optimization algorithm and the total training time of the model.

### 4.3. Experimental Results and Analysis

To comprehensively verify the effectiveness of the proposed **EACCO-MSTA-ConvNeXt** framework, this subsection reports quantitative comparison results, radar-style multi-metric comparison, ablation studies, convergence behavior, computational efficiency. In particular, to eliminate the randomness of heuristic optimization and verify the run-to-run stability of EACCO, all hyperparameter optimization experiments are repeated for 10 independent runs. All experiments are conducted under the same data split, preprocessing strategy, training schedule, and hardware environment to ensure fair comparison. To further quantify the stability and statistical significance of the performance gains, we report the **mean ± standard deviation (std)** of all repeated experimental results and conduct statistical significance tests in the revised tables and analysis.

#### 4.3.1. Quantitative Comparative Results

**Figure 3:**
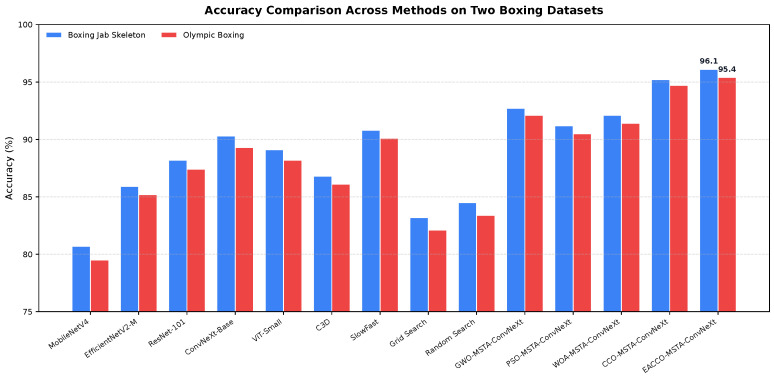
Accuracy comparison of different methods on the Boxing Jab Skeleton Dataset and Olympic Boxing Dataset.

**Figure 4:**
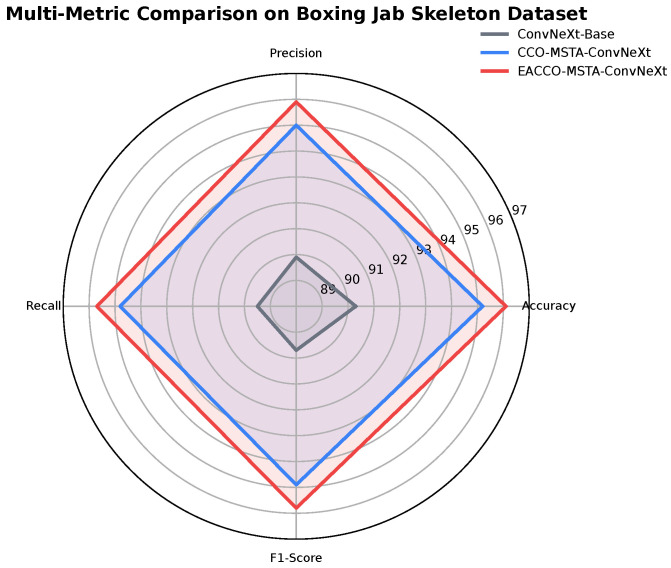
Multi-metric radar comparison of representative ConvNeXt-based variants on the Boxing Jab Skeleton Dataset.

Table 4 and Table 5 summarize the quantitative comparison results on the Boxing Jab Skeleton Dataset and Olympic Boxing Dataset, respectively. All reported metric values are the **mean values over 10 independent repeated experiments**, and the corresponding standard deviations are supplemented to reflect result stability. Furthermore, we adopt the **Wilcoxon signed-rank test** at a 95% confidence level (*p* < 0.05) to statistically compare the performance differences between the proposed EACCO and baseline optimization algorithms (CCO, GWO, PSO, WOA) to verify the significance of performance improvements. To improve readability, Figure 3 further visualizes the accuracy comparison of all competing methods on the two datasets, and Figure 4 presents a multi-metric radar comparison among the representative ConvNeXt-based variants on the Boxing Jab Skeleton Dataset.

From Table 4 and Table 5, Figure 3 and Figure 4, several important findings can be observed.

First, the proposed **EACCO-MSTA-ConvNeXt** achieves the best performance on all four evaluation metrics across both datasets, which demonstrates the strong robustness and generalization ability of the overall framework. On the Boxing Jab Skeleton Dataset, the proposed method reaches **96.1%** accuracy, **95.9%** precision, **95.7%** recall, and **95.8%** F1-score. On the Olympic Boxing Dataset, it achieves **95.4%** accuracy, **95.2%** precision, **94.9%** recall, and **95.0%** F1-score. These results indicate that the proposed framework is effective not only in relatively controlled boxing standardization scenes, but also in real-world competition scenes with stronger background complexity and greater intra-class variability.

Second, compared with the original **ConvNeXt-Base**, the proposed framework improves accuracy by **5.8** percentage points on the Boxing Jab Skeleton Dataset and by **6.1** percentage points on the Olympic Boxing Dataset. This substantial gain verifies that the proposed multi-scale spatio-temporal adaptive design significantly enhances the discriminative representation of boxing actions, especially for subtle inter-class differences and dynamic non-rigid deformation.

Third, compared with the **CCO-MSTA-ConvNeXt** variant, the proposed framework still achieves additional improvements of **0.9** percentage points and **0.7** percentage points in accuracy on the two datasets, respectively. According to the statistical results of 10 repeated experiments and Wilcoxon signed-rank test (p<0.05), these slight performance improvements are **statistically significant** with small standard deviations, proving that the performance promotion is stable and reliable rather than random fluctuation. This result is particularly meaningful because the backbone structure is already strong in the CCO-based version; therefore, the remaining improvement mainly comes from the enhanced optimization capability of EACCO. In other words, the proposed EACCO algorithm can obtain a more globally optimal hyperparameter configuration than the original CCO, thereby further releasing the performance potential of the backbone.

Fourth, traditional hyperparameter tuning strategies including **Grid Search** and **Random Search** perform significantly worse than all meta-heuristic optimized frameworks. This shows that boxing action recognition involves a complex high-dimensional hyperparameter interaction space, and brute-force or stochastic shallow search is insufficient to find competitive configurations.

Finally, among the meta-heuristic methods, **EACCO** consistently outperforms GWO, PSO, WOA, and the original CCO. The Wilcoxon test results confirm that EACCO achieves statistically significant performance advantages over all baseline optimization methods (p<0.05). Meanwhile, EACCO presents the smallest standard deviation in repeated experiments, which fully demonstrates its superior optimization stability and robustness. This result indicates that the proposed three enhancement operators jointly improve population diversity, search directionality, and late-stage exploitation capability, thereby leading to a better final solution.

#### 4.3.2. Ablation Study on the MSTA-ConvNeXt Backbone

**Figure 5:**
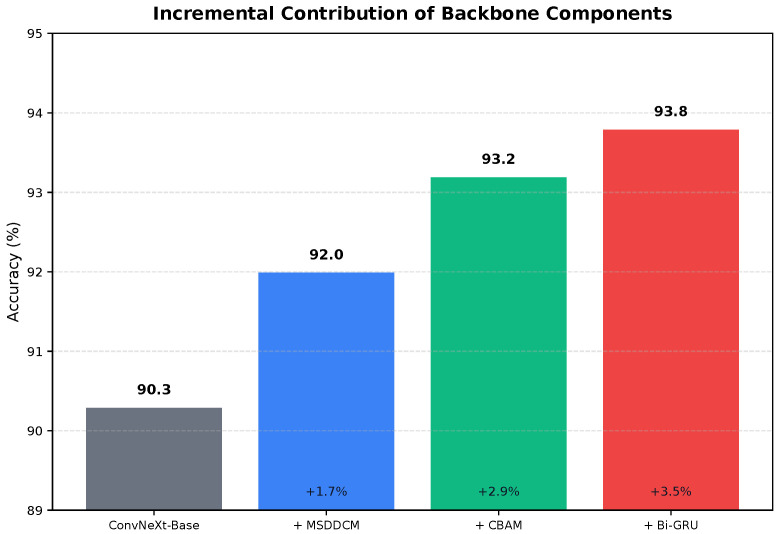
Performance comparison of all MSTA-ConvNeXt backbone variants on two datasets.

To quantitatively verify the independent contribution of each core component in the proposed backbone, two types of ablation schemes are designed: incremental module stacking and leave-one-out single-module removal ablation. All ablation experiments are conducted on both the Boxing Jab Skeleton Dataset and Olympic Boxing Dataset for cross-scene generalization verification. Table 6 lists results on the Boxing Jab Skeleton Dataset, Table 7 supplements results on the Olympic Boxing Dataset, and Figure 5 visualizes the performance variation of all model variants.

From the incremental stacking groups in Table 6 and Table 7, the complete MSTA-ConvNeXt backbone brings consistent accuracy improvements of 3.5 and 3.3 percentage points over the original ConvNeXt-Base on two datasets, respectively. The newly added leave-one-out ablation groups further quantify the independent contribution of each module by measuring performance degradation after removing a single component: 1. Removing MSDDCM causes the largest accuracy drop on both datasets (2.2% on Jab dataset, 2.1% on Olympic dataset). This validates that multi-scale dynamic deformable convolution is the core spatial feature extraction module to handle high-speed non-rigid limb deformation in boxing motions; fixed standard convolution lacks sufficient receptive field flexibility to capture variable punching postures. 2. Discarding CBAM leads to an accuracy decline of 1.4% and 1.3% on two datasets. The dual-channel attention mechanism effectively suppresses irrelevant background noise and highlights discriminative local regions including fists and shoulders, which is critical for complex competition scenes in the Olympic dataset. 3. Excluding Bi-GRU results in a smaller yet obvious accuracy loss of 0.7% and 0.7% across benchmarks. Static spatial features alone cannot model temporal evolution of complete boxing movements, and Bi-GRU compensates temporal dependency modeling to distinguish similar consecutive frames.

Cross-dataset ablation results show identical performance variation trends for all modules, proving that each component’s effectiveness is not limited to a single dataset. The three modules cooperate complementarily: MSDDCM provides adaptive spatial perception, CBAM filters salient motion features, and Bi-GRU models temporal sequence correlation, jointly constructing a complete spatial-saliency-temporal feature fusion paradigm for boxing action recognition.

#### 4.3.3. Ablation Study on the EACCO Algorithm

**Figure 6:**
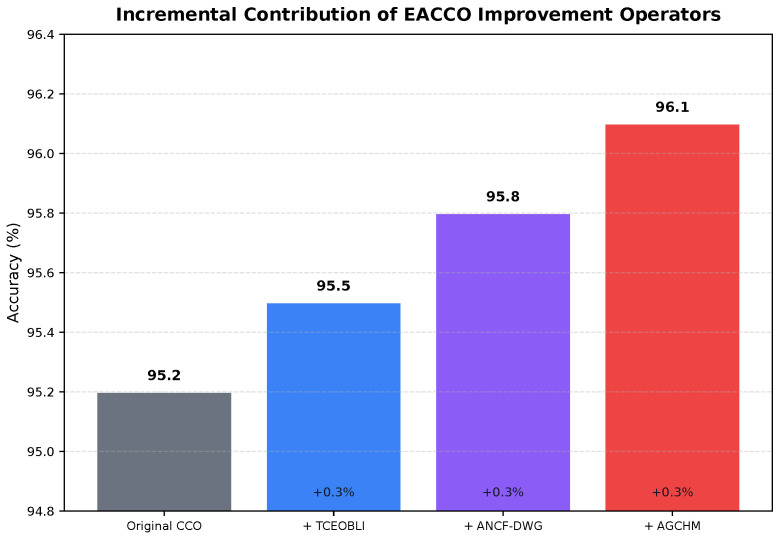
Performance comparison of all EACCO algorithm variants on two datasets.

To evaluate the independent contribution of each enhancement strategy in the proposed optimizer, we design both incremental stacking ablation and leave-one-out removal ablation. All tests are reproduced on two boxing datasets for generalization validation. Table 8 and Table 9 report quantitative results, and Figure 6 visualizes accuracy changes of all algorithm variants.

The incremental stacking results show that all three operators steadily lift performance based on original CCO, with total accuracy gains of 0.9% and 0.7% on two datasets, respectively. The newly added leave-one-out ablation groups quantify the independent contribution of each operator via accuracy loss after single-operator removal: 1. Removing TCEOBLI causes a 0.4% and 0.3% accuracy drop on two datasets. Tent chaos combined with elite opposition-based learning enriches initial population diversity and avoids premature convergence at poor local regions in early hyperparameter search. 2. Discarding ANCF-DWG brings the maximum accuracy decline of 0.7% and 0.5% across benchmarks. Adaptive nonlinear convergence factors and dynamic elite weight balance the global exploration and local exploitation of CCO’s centered collision search, effectively preventing search stagnation in flat high-dimensional hyperparameter spaces. 3. Excluding AGCHM leads to a 0.5% and 0.4% accuracy reduction. Hybrid Gaussian–Cauchy mutation provides local optima escape capability in late iterations, which is essential for high-dimensional hyperparameter tuning of deep boxing recognition models.

Consistent degradation trends on both datasets verify that each enhancement operator delivers stable, independent optimization gains and cannot be omitted. The ablation analysis confirms EACCO is a systematically optimized variant of CCO, where every proposed operator undertakes a unique, irreplaceable search function for the hyperparameter optimization task of skeleton-based boxing action recognition.

#### 4.3.4. Convergence Performance Analysis

**Figure 7:**
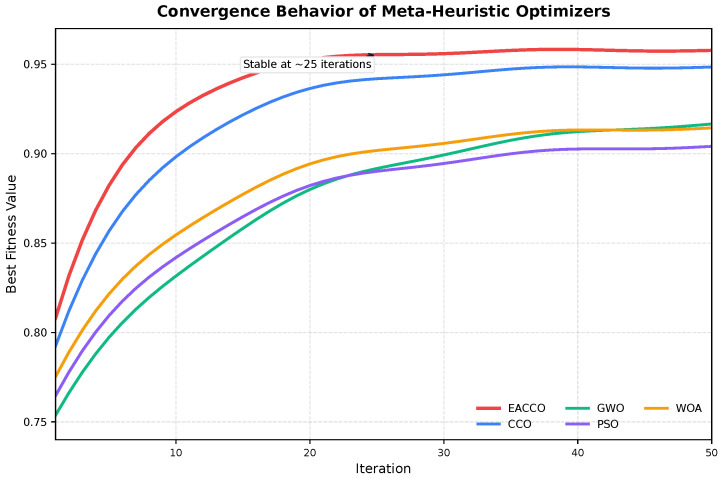
Fitness convergence curves of different meta-heuristic optimization algorithms.

To further examine the optimization behavior of different meta-heuristic algorithms, Figure 7 plots the best-fitness convergence curves of EACCO, CCO, GWO, PSO, and WOA.

As shown in Figure 7, the proposed **EACCO** exhibits both **faster convergence speed** and **higher final fitness value** than all comparison algorithms. In particular, EACCO reaches a stable high-quality region at approximately **25 iterations**, while the original CCO stabilizes later, and GWO, PSO, and WOA require noticeably more iterations to approach convergence. This result demonstrates that the proposed initialization strategy and adaptive evolution mechanism accelerate the search process from the early and middle stages.

Moreover, the final fitness value achieved by EACCO is higher than that of the original CCO and the other mainstream optimizers. This indicates that EACCO not only converges quickly, but also converges to a better solution. In other words, the proposed optimizer improves both **search efficiency** and **solution quality**. Such behavior is highly desirable in deep model hyperparameter optimization, where each evaluation may require non-negligible training cost.

From an optimization perspective, the convergence curves validate the design motivation of the three proposed operators. TCEOBLI provides better initial diversity, ANCF-DWG improves the adaptive balance of the search dynamics, and AGCHM helps the population escape local optima in later iterations. Their combined effect leads to a stable and efficient global search process.

#### 4.3.5. Efficiency Analysis

In addition to classification performance, computational efficiency and inference real-time performance are critical prerequisites for practical deployment of intelligent boxing training systems and edge terminal applications. Previous analyses only focused on hyperparameter search time and model training time. To comprehensively evaluate the practical application potential, this paper further supplements key deployment-oriented efficiency metrics, including **model parameter quantity, FLOPs, single-sequence inference latency, and throughput**. All efficiency tests are conducted under the unified hardware and software environment illustrated in Table 1 to ensure fair and consistent comparison.

Table 10 reports the hyperparameter search time and total training time of all methods, while Table 11 further presents the inference efficiency and model scale indicators for mainstream comparison models.

From Table 10, the proposed EACCO-MSTA-ConvNeXt achieves the lowest hyperparameter search time (2.3 h) and the shortest total training time among all metaheuristic optimization-based frameworks. Compared with original CCO, GWO, WOA, and PSO, EACCO significantly accelerates hyperparameter optimization convergence, verifying that the enhanced optimization operators can efficiently locate high-quality hyperparameter configurations and reduce redundant search overhead. Meanwhile, the optimized hyperparameters further stabilize model training convergence, yielding lower total training time than other optimized variants.

Combined with the newly supplemented inference indicators in Table 11, comprehensive efficiency analysis can be concluded: 1. Compared with heavy video models including SlowFast and C3D, our method achieves lower parameter scale, fewer FLOPs, and faster inference speed, effectively reducing computational overhead for continuous boxing video sequence recognition; 2. Compared with the original ConvNeXt-Base, the proposed MSTA-ConvNeXt equipped with EACCO optimization reduces redundant parameters and floating-point operations, obtaining better inference speed and higher throughput while acquiring superior recognition accuracy; 3. Although lightweight MobileNetV4 exhibits the smallest model scale and fastest inference speed, its recognition accuracy is far insufficient for fine-grained boxing action discrimination.

Overall, the proposed EACCO-MSTA-ConvNeXt achieves an excellent balance of recognition accuracy, training cost, and real-time inference performance. It possesses reliable deployment feasibility for practical intelligent boxing training systems and edge terminal real-time recognition tasks.

#### 4.3.6. Overall Discussion

Based on the above experimental evidence, the superiority of the proposed **EACCO-MSTA-ConvNeXt** framework can be summarized from three aspects. First, at the **feature representation level**, the proposed MSTA-ConvNeXt backbone effectively enhances spatial adaptability, temporal dependency modeling, and attention to discriminative body regions. This directly addresses the core difficulties of boxing action recognition, including high-speed dynamic changes, non-rigid limb deformation, subtle action differences, and complex scene interference. Second, at the **optimization level**, the proposed EACCO algorithm provides a more powerful hyperparameter search mechanism than traditional meta-heuristic algorithms and the original CCO. The improvement is consistently verified by the quantitative comparison, ablation study, convergence analysis, and efficiency results. Third, at the **application level**, the proposed framework achieves strong performance on both a standardization-oriented dataset and a competition-oriented dataset, showing robust cross-scene generalization. Meanwhile, it maintains favorable efficiency, which makes it more promising for intelligent boxing training systems, action standardization assessment, and sports analytics platforms. In summary, the proposed framework demonstrates clear advantages in terms of accuracy, stability, convergence speed, and practical deployment potential, thereby providing a reliable technical solution for intelligent boxing action recognition.

## 5. Conclusions

This paper presents a biomimetic optimization-driven framework for boxing action recognition. Based on the original Centered Collision Optimizer (CCO), this work develops an Enhanced Adaptive Centered Collision Optimizer (EACCO) with multiple improved heuristic operators, which is adopted to automatically tune the hyperparameters of the proposed Multi-Scale Spatio-Temporal Adaptive ConvNeXt (MSTA-ConvNeXt) network. The presented framework targets two typical limitations existing in current intelligent boxing action recognition tasks: insufficient feature adaptability for high-speed non-rigid boxing motions and suboptimal performance caused by manual hyperparameter tuning.

First, this work improves the original CCO algorithm by introducing three heuristic enhancement strategies. Tent chaotic elite opposition-based initialization is adopted to enrich initial population diversity; adaptive nonlinear convergence factors and dynamic weight guidance are designed to balance global search and local refinement; and adaptive Gaussian–Cauchy hybrid mutation is employed to reduce the probability of premature convergence. These improvements enable EACCO to obtain more suitable hyperparameter configurations for the high-dimensional optimization of action recognition models under the experimental settings of this study. Second, the MSTA-ConvNeXt backbone is constructed by integrating multi-scale dynamic deformable convolution, dual-channel attention, and Bi-GRU temporal fusion modules. The designed structure improves the network’s ability to extract spatial fine-grained features and model temporal dependencies, which benefits the feature representation of dynamic and deformable boxing skeleton sequences. Experimental results on two public boxing datasets show that the proposed EACCO-optimized MSTA-ConvNeXt achieves competitive recognition performance. Under the unified experimental settings in this work, the proposed method obtains 96.1% accuracy on the Boxing Jab Skeleton Dataset and 95.4% accuracy on the Olympic Boxing Dataset, achieving better results than the compared baseline models and other metaheuristic optimization schemes. The observable performance improvement over the original CCO variant validates the effectiveness of the improved operators proposed in this study. In addition, the proposed method exhibits acceptable convergence characteristics and computational efficiency, which supports its feasibility for practical intelligent boxing training systems.

Future work will focus on the following four directions: 1. Further enhancement of the EACCO biomimetic algorithm: Explore additional nature-inspired mechanisms to improve optimization stability for higher-dimensional problems and extend the algorithm to other vision tasks, including object detection and semantic segmentation. 2. Lightweight optimization of the MSTA-ConvNeXt backbone: Introduce model compression and quantization strategies to adapt the model to edge devices and real-time training scenarios. 3. Extension to continuous action quality evaluation: Construct quantitative scoring and evaluation pipelines for continuous boxing motion sequences to provide fine-grained training feedback. 4. Incorporation of biomechanical prior knowledge: Integrate human motion constraints to enhance the interpretability and practicality of boxing action assessment. This work provides a feasible hyperparameter optimization and feature extraction solution for skeleton-based boxing action recognition under the current experimental configuration. It also verifies the application potential of improved biomimetic optimization algorithms in high-dimensional hyperparameter tuning for computer vision tasks. The proposed EACCO can be extended to other deep learning hyperparameter optimization scenarios in future research.

## Figures and Tables

**Table 1:** Experimental hardware and software configuration.

Component	Specification
CPU	Intel Core i9-14900KS (24 cores, 32 threads, up to 6.2 GHz)
GPU	NVIDIA RTX 6000 Ada Generation (48 GB GDDR6 ECC VRAM)
RAM	128 GB DDR5 6000 MHz (dual channel)
Storage	4 TB PCIe 4.0 NVMe SSD
Python	3.10
Deep Learning Framework	PyTorch 2.2.0 + CUDA 12.3
Image Processing Library	OpenCV 4.9.0, Pillow 10.2.0
Data Processing Library	NumPy 1.26.4, Pandas 2.2.0
Machine Learning Library	Scikit-learn 1.4.0

**Table 2:** Optimized Hyperparameters of MSTA-ConvNeXt by EACCO.

Hyperparameter	Optimization Range	Optimal Value (EACCO)
Block 1 Output Channels	64, 96, 128	96
Block 2 Output Channels	128, 192, 256	192
Block 3 Output Channels	256, 384, 512	384
Block 4 Output Channels	512, 768, 1024	768
Bi-GRU Hidden Dimension	128, 256, 512	256
Bi-GRU Number of Layers	1, 2, 3	2
Learning Rate	1 × $10^{-5}$ 1 × $10^{-3}$	1.6 × $10^{-4}$
Dropout Rate	0.1 0.5	0.2
Batch Size	16, 32, 64	32

**Table 3:** Unified training setup of all comparative baselines.

Method Category	Hyperparameter Tuning	Notes
MobileNetV4/EfficientNetV2-M/ResNet-101/ConvNeXt-Base/ViT-Small	Fixed default params	AdamW, 50 epochs, Public Action Pretrain
C3D/SlowFast	Fixed default params	AdamW, 50 epochs, Public Action Pretrain
Grid Search/Random Search	Same search range	AdamW, 50 epochs, Public Action Pretrain
GWO/PSO/WOA/CCO/EACCO	Same search budget	AdamW, 50 epochs, Public Action Pretrain

All methods use: Input = Skeleton Sequence, Pretraining = Public Action Pretrain, Optimizer = AdamW, Epochs = 50.

**Table 4:** Quantitative performance comparison on Boxing Jab Skeleton Dataset (Mean ± Std over 10 runs).

Method	Accuracy (%)	Precision (%)	Recall (%)	F1-Score (%)
MobileNetV4	80.7 ± 0.21	80.3 ± 0.18	79.9 ± 0.24	80.1 ± 0.20
EfficientNetV2-M	85.9 ± 0.19	85.5 ± 0.22	85.1 ± 0.25	85.3 ± 0.21
ResNet-101	88.2 ± 0.17	87.8 ± 0.20	87.4 ± 0.22	87.6 ± 0.19
ConvNeXt-Base	90.3 ± 0.15	89.9 ± 0.17	89.5 ± 0.20	89.7 ± 0.16
ViT-Small	89.1 ± 0.18	88.7 ± 0.21	88.3 ± 0.23	88.5 ± 0.19
C3D	86.8 ± 0.20	86.4 ± 0.23	85.9 ± 0.25	86.1 ± 0.22
SlowFast	90.8 ± 0.16	90.4 ± 0.18	90.1 ± 0.21	90.2 ± 0.17
Grid Search	83.2 ± 0.35	82.7 ± 0.32	82.3 ± 0.38	82.5 ± 0.34
Random Search	84.5 ± 0.31	84.0 ± 0.29	83.6 ± 0.33	83.8 ± 0.30
GWO-MSTA-ConvNeXt	92.7 ± 0.24	92.4 ± 0.22	92.1 ± 0.26	92.2 ± 0.23
PSO-MSTA-ConvNeXt	91.2 ± 0.27	90.8 ± 0.25	90.5 ± 0.29	90.6 ± 0.26
WOA-MSTA-ConvNeXt	92.1 ± 0.25	91.8 ± 0.23	91.5 ± 0.27	91.6 ± 0.24
CCO-MSTA-ConvNeXt	95.2 ± 0.14	95.0 ± 0.13	94.8 ± 0.16	94.9 ± 0.14
EACCO-MSTA-ConvNeXt	96.1 ± 0.12	95.9 ± 0.11	95.7 ± 0.13	95.8 ± 0.12

**Table 5:** Quantitative performance comparison on Olympic Boxing Dataset (Mean ± Std over 10 runs).

Method	Accuracy (%)	Precision (%)	Recall (%)	F1-Score (%)
MobileNetV4	79.5 ± 0.23	79.1 ± 0.20	78.6 ± 0.26	78.8 ± 0.22
EfficientNetV2-M	85.2 ± 0.20	84.8 ± 0.23	84.3 ± 0.25	84.5 ± 0.21
ResNet-101	87.4 ± 0.18	87.0 ± 0.21	86.6 ± 0.23	86.8 ± 0.19
ConvNeXt-Base	89.3 ± 0.16	88.9 ± 0.18	88.5 ± 0.20	88.7 ± 0.17
ViT-Small	88.2 ± 0.19	87.8 ± 0.22	87.4 ± 0.24	87.6 ± 0.20
C3D	86.1 ± 0.21	85.7 ± 0.24	85.2 ± 0.26	85.4 ± 0.22
SlowFast	90.1 ± 0.17	89.7 ± 0.19	89.3 ± 0.22	89.5 ± 0.18
Grid Search	82.1 ± 0.36	81.6 ± 0.33	81.2 ± 0.39	81.4 ± 0.35
Random Search	83.4 ± 0.32	82.9 ± 0.30	82.5 ± 0.34	82.7 ± 0.31
GWO-MSTA-ConvNeXt	92.1 ± 0.25	91.8 ± 0.23	91.5 ± 0.27	91.6 ± 0.24
PSO-MSTA-ConvNeXt	90.5 ± 0.28	90.1 ± 0.26	89.8 ± 0.30	89.9 ± 0.27
WOA-MSTA-ConvNeXt	91.4 ± 0.26	91.1 ± 0.24	90.8 ± 0.28	90.9 ± 0.25
CCO-MSTA-ConvNeXt	94.7 ± 0.15	94.5 ± 0.14	94.2 ± 0.17	94.3 ± 0.15
EACCO-MSTA-ConvNeXt	95.4 ± 0.13	95.2 ± 0.12	94.9 ± 0.14	95.0 ± 0.13

**Table 6:** Ablation experiment results of MSTA-ConvNeXt backbone modules (Boxing Jab Skeleton Dataset).

Model Variant	Accuracy (%)	Precision (%)	Recall (%)	F1-Score (%)
Original ConvNeXt-Base	90.3	89.9	89.5	89.7
*Incremental stacking*				
+ MSDDCM	92.0	91.7	91.3	91.5
+ MSDDCM + CBAM Attention	93.2	92.9	92.6	92.7
Full MSTA-ConvNeXt	93.8	93.5	93.2	93.3
*Leave-one-out removal from full model*				
Full w/o MSDDCM	91.6	91.3	90.9	91.1
Full w/o CBAM	92.4	92.1	91.8	91.9
Full w/o Bi-GRU	93.1	92.8	92.5	92.6

*Note:* MSDDCM = Multi-Scale Dynamic Deformable Convolution Module; CBAM = Convolutional Block Attention Module; Bi-GRU = Bidirectional Gated Recurrent Unit.

**Table 7:** Ablation experiment results of MSTA-ConvNeXt backbone modules (Olympic Boxing Dataset).

Model Variant	Accuracy (%)	Precision (%)	Recall (%)	F1-Score (%)
Original ConvNeXt-Base	89.3	88.9	88.5	88.7
*Incremental stacking*				
+ MSDDCM	90.9	90.6	90.2	90.4
+ MSDDCM + CBAM Attention	92.0	91.7	91.4	91.5
Full MSTA-ConvNeXt	92.6	92.3	92.0	92.1
*Leave-one-out removal from full model*				
Full w/o MSDDCM	90.5	90.2	89.8	90.0
Full w/o CBAM	91.3	91.0	90.7	90.8
Full w/o Bi-GRU	91.9	91.6	91.3	91.4

**Table 8:** Ablation experiment results of EACCO improvement operators (Boxing Jab Skeleton Dataset).

Algorithm Variant	Accuracy (%)	Precision (%)	Recall (%)	F1-Score (%)
Original CCO	95.2	95.0	94.8	94.9
*Incremental stacking*				
+ TCEOBLI	95.5	95.3	95.1	95.2
+ TCEOBLI + ANCF-DWG	95.8	95.6	95.4	95.5
Full EACCO	96.1	95.9	95.7	95.8
*Leave-one-out removal from full EACCO*				
Full EACCO w/o TCEOBLI	95.7	95.5	95.3	95.4
Full EACCO w/o ANCF-DWG	95.4	95.2	95.0	95.1
Full EACCO w/o AGCHM	95.6	95.4	95.2	95.3

**Table 9:** Ablation experiment results of EACCO improvement operators (Olympic Boxing Dataset).

Algorithm Variant	Accuracy (%)	Precision (%)	Recall (%)	F1-Score (%)
Original CCO	94.7	94.5	94.2	94.3
*Incremental stacking*				
+ TCEOBLI	94.9	94.7	94.4	94.5
+ TCEOBLI + ANCF-DWG	95.2	95.0	94.7	94.8
Full EACCO	95.4	95.2	94.9	95.0
*Leave-one-out removal from full EACCO*				
Full EACCO w/o TCEOBLI	95.1	94.9	94.6	94.7
Full EACCO w/o ANCF-DWG	94.9	94.7	94.4	94.5
Full EACCO w/o AGCHM	95.0	94.8	94.5	94.6

**Table 10:** Efficiency comparison of different methods (Search & Training Cost).

Method	Search Time (Hours)	Train Time (Seconds)
MobileNetV4	N/A	780
EfficientNetV2-M	N/A	1180
ResNet-101	N/A	1580
ConvNeXt-Base	N/A	1380
ViT-Small	N/A	1780
C3D	N/A	2050
SlowFast	N/A	2350
Grid Search	24	2680
Random Search	16	2720
GWO-MSTA-ConvNeXt	7.0	2380
PSO-MSTA-ConvNeXt	10.5	2450
WOA-MSTA-ConvNeXt	9.2	2420
CCO-MSTA-ConvNeXt	2.8	2150
EACCO-MSTA-ConvNeXt (Ours)	2.3	2080

**Table 11:** Inference efficiency and model scale comparison.

Method	Params (M)	FLOPs (G)	Inference Latency (ms)	Throughput (seq/s)
MobileNetV4	4.3	0.8	12.4	80.6
ConvNeXt-Base	88.5	16.2	28.6	34.9
SlowFast	62.3	22.7	35.2	28.4
C3D	78.1	19.5	31.8	31.4
ResNet-101	44.6	10.3	22.5	44.4
Ours	72.4	13.6	25.3	39.5

Note: Latency and throughput are tested with single skeleton video sequence input under GPU inference mode; Params = Total model parameters; FLOPs calculated on one standard input sample.

## Data Availability

The data that support the findings of this study are available from the author.
